# Stuck Below: Failure of Tooth Eruption and Hereditary Enamel Defects

**DOI:** 10.1016/j.identj.2025.100942

**Published:** 2025-08-05

**Authors:** Piranit Nik Kantaputra, Thamon Sirikrai, Jeremy Green

**Affiliations:** a Center of Excellence in Medical Genetics Research, Faculty of Dentistry, Chiang Mai University, Chiang Mai, Thailand; b Division of Pediatric Dentistry, Department of Orthodontics and Pediatric Dentistry, Faculty of Dentistry, Chiang Mai University, Chiang Mai, Thailand; c Centre for Craniofacial Regeneration and Biology, King's College London, Guy's Hospital, London, UK

**Keywords:** Enamel malformation, Delay of tooth eruption, Tooth eruption failure, Enamel defects, Enamel anomalies

Recently, one of us (PK) reported a patient with compound heterozygous mutations in the *PEX1* gene who was diagnosed with Heimler syndrome—an autosomal recessive multisystem disorder characterised by amelogenesis imperfecta (AI), sensorineural hearing loss, and retinitis pigmentosa.^1^ Literature search revealed that several patients with syndromic or non-syndromic (isolated) AI also exhibited delay or failure of tooth eruption (Figure 1, Table 1). The patient with Heimler syndrome demonstrated eruption failure secondary to ankylosis, which was demonstrated by cone beam computed tomography.^1^ The observation of failure of tooth eruption in patients with AI raises the notion that successful eruption of teeth may be intrinsically linked to proper enamel formation, suggesting that the process of amelogenesis, governed by enamel-specific genes and pathways, may play a more active role in coordinating eruption than previously thought.

**Fig. 1:**
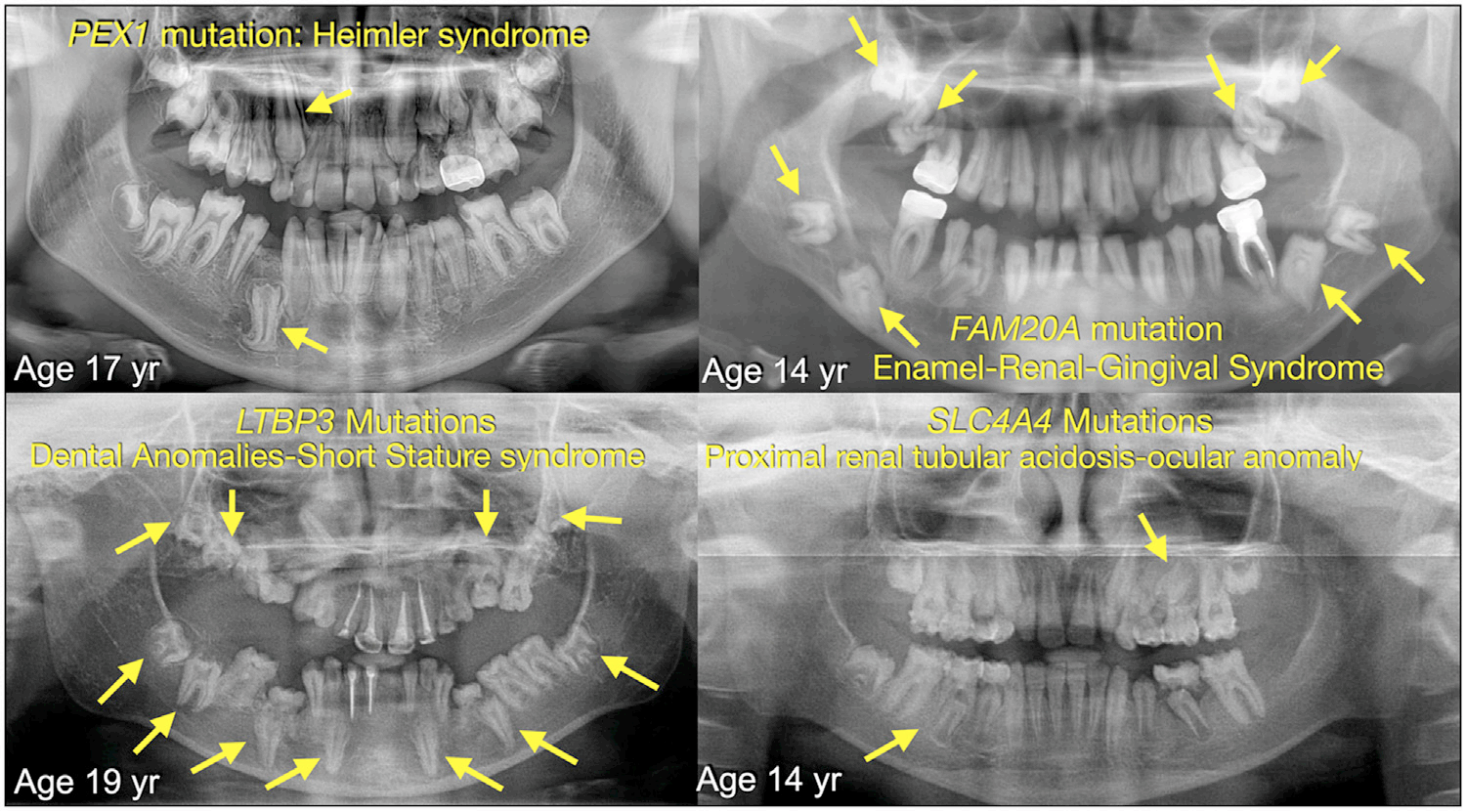
Failure of tooth eruption in patients with amelogenesis imperfecta.

**Table 1:** Amelogenesis-associated genes which have been reported to be associated with eruption disturbance.

Genes	Functions of the genes	Isolated/syndromic	Hypoplasia/Hypocalcification/Hypomaturation/Hypomineralisation Types of AI	Eruption disturbance	References
*ENAM* (MIM 606585)	ENAMELIN initiates and guides enamel crystal growth	Isolated	Hypoplasia type	Failure of tooth eruption	Lindemeyer et al., 2010; Hart et al., 2003 ^13^^,^^14^
*AMELX* (MIM *300391)	AMELOGENIN regulates enamel crystal growth, orientation, and spacing during amelogenesis.	Isolated	Hypoplasia/Hypocalcification/Hypomaturation Types	Failure and delay of tooth eruption	Leban et al., 2022; Wang et al., 2024 ^15^^,^^16^
ODAPH (MIM 614829)	ODAPH promotes enamel crystal maturation and organization.	Isolated	Hypoplasia/Hypocalcification/Hypomaturation Types	Failure of tooth eruption	Wang et al., 2024 ^17^
FAM83H (MIM 611927)	FAM83H plays a critical role in the organization and mineralization of enamel by influencing the structure of the enamel matrix and the behaviour of ameloblasts.	Isolated	Hypocalcification type	Failure and delay of tooth eruption	Wang et al., 2021; Parry et al., 2012 ^18^^,^^19^
*RELT* (MIM 611211)	RELT plays a role in cell signalling during enamel formation. It is expressed in ameloblasts and is essential for proper enamel mineralisation and structure.	Isolated	Hypoplasia/hypomineralisation types	Failure of tooth eruption	Nikolopoulos et al., 2020 ^20^
*WDR72* (MIM 613214)	WDR72 regulates vesicle trafficking and acid-base balance necessary for enamel mineralization.	Isolated	Hypomineralisation/Hypomaturation type	Delay of tooth eruption	Katsura et al., 2014^21^
*KLK4* (MIM 603767)	KLK4 breaks down enamel matrix proteins to remove organic material, enabling enamel to harden and mineralise properly.	Isolated	Hypomaturation type	Failure and delay of tooth eruption	Wang et al., 2013^22^
*SLC4A4* (MIM 603345)	SLC4A4 functions as a major pH regulator and bicarbonate transporter. It is crucial for maintaining optimal physiological pH in the extracellular matrix during the secretory and maturation stages of enamel formation.	Proximal renal tubular acidosis-ocular anomaly syndrome (PRTAO; MIM 604278)	Hypocalcification type	Delay of tooth eruption	Kantaputra et al., 2022 ^8^
*PEX1* (MIM 602136)	The PEX1 is involved in peroxisome biogenesis, essential for normal cellular metabolism. Proper peroxisome activity is important for lipid metabolism and detoxification, which indirectly affects enamel matrix production and mineralisation	Heimler syndrome (HMLR1; MIM 234580)	Hypoplasia type	Failure of tooth eruption	Kantaputra et al., 2025 ^1^
*FAM20A* (MIM 611062)	FAM20A, a regulatory protein, that forms a complex with FAM20C to control the phosphorylation of enamel matrix proteins. This phosphorylation is essential for proper enamel mineralisation and structural organisation.	Enamel-Renal-Gingival syndrome (ERG; MIM 204690)	Hypoplasia type	Failure of tooth eruption	Kantaputra et al., 2014a; 2014b; Kantaputra 2017 2, 3, 4
*LTBP3* (MIM 602090)	LTBP3 is essential for proper ameloblast differentiation and enamel matrix production.	Dental Anomalies Short Stature syndrome (DASS; MIM 601216)	Hypoplasia type	Failure of tooth eruption	Huckert et al., 2015; Kantaputra et al., 2022 ^6^^,^^7^
*DLX3* (MIM 600525)	DLX3 is a transcription factor that regulates ameloblast development and enamel protein expression, ensuring proper enamel formation and mineralisation.	Trichodontoosseous syndrome (TDO; MIM 190320)	Hypoplasia/ hypocalcification /hypomaturation type	Delayed or failure of tooth eruption/Precoccious tooth eruption	Islam et al., 2005; Jain et al., 2017; Whitehouse et al., 2019; Bonnet et al., 2020 9, 10, 11, 12

## Syndromic and non-syndromic failure of tooth eruption

Failure of tooth eruption can occur in both syndromic and non-syndromic contexts and may be associated with either normal or abnormal enamel. Syndromic forms of failure of tooth eruption with enamel defects are well documented and include *FAM20A*-associated enamel–renal–gingival syndrome (ERG; MIM 204690),^2^, ^3^, ^4^ *PEX1*-associated Heimler syndrome (HMLR1; MIM 234580),^5^ *LTBP3*-associated dental anomalies and short stature syndrome (DASS; MIM 601216),^6^^,^^7^ *SLC4A4*-associated proximal renal tubular acidosis–ocular anomaly syndrome (PRTAO; MIM 604278)^8^ and *DLX3*-associated tricho-dento-osseous syndrome (TDO; MIM 190320) (Table 1).^9^, ^10^, ^11^, ^12^

In addition to these syndromic conditions, failure of tooth eruption can also occur in patients with isolated forms of AI. Isolated AI with associated failure of tooth eruption has been linked to pathogenic variants in genes such as *ENAM* (MIM 606585),^13^^,^^14^ *AMELX* (MIM 300391),^15^^,^^16^ *ODAPH* (MIM 614829),^17^ *FAM83H* (MIM 611927),^18^^,^^19^ *RELT* (MIM 611211),^20^ *WDR72* (MIM 613214)^21^ and *KLK4* (MIM 603767) (Table 1).^22^

## Failure of tooth eruption in patients with normal enamel

Intriguingly, failure of tooth eruption is not always a consequence of defective enamel. Several well-characterized syndromic and isolated conditions demonstrate that tooth eruption can fail entirely despite perfectly normal enamel structure. For example, primary failure of eruption (PFE; MIM 125350) results from pathogenic variants in *PTH1R* (MIM 168468) and disrupts the eruption pathway without affecting enamel mineralisation.^23^ Cleidocranial dysplasia (CLCD1; MIM 119600), caused by *RUNX2* (MIM 600211) mutations, is another classic example: multiple impacted supernumerary teeth arise alongside abnormal skeletal development rather than enamel malformation.^24^ Trichorhinophalangeal syndrome type I (TRPS1; MIM 190350), associated with *TRPS1* (MIM 604386) mutations, may also present with eruption failure of multiple supernumerary teeth while preserving enamel integrity.^25^, ^26^, ^27^ Likewise, *APC*-associated familial adenomatous polyposis, or Gardner syndrome (FAP1; MIM 175100), often includes multiple unerupted supernumerary teeth together with jaw osteomas and odontomas but not enamel defects.^28^ Moreover, *CLCN7*-associated infantile malignant autosomal recessive osteopetrosis (ARO; MIM 259700) illustrates how dense, sclerotic bone can physically entrap developing teeth, preventing eruption despite normal enamel.^29^ Lastly, the failure of tooth eruption in patients with *ANTXR1*-associated GAPO syndrome (GAPOS; MIM 230740), who have normal enamel, likely results from abnormal connective tissue remodelling and impaired bone resorption rather than from a primary defect in enamel formation.^30^^,^^31^ Taken together, these conditions highlight that failure of tooth eruption can result from diverse mechanisms—ranging from disrupted eruption pathways and excessive bone density to supernumerary teeth and local physical obstructions—rather than from defects in the enamel itself.

## How teeth erupt

Tooth eruption is a complex, precisely regulated biological process that governs the precise movement of developing tooth germs from their initial positions within the jawbone to their final and functional locations in the oral cavity.^32^, ^33^, ^34^ This process necessitates coordinated interactions among multiple components, including the integrity of the dental follicle,^34^ the activity of osteoclasts responsible for bone resorption,^35^ and the involvement of key molecular signalling pathways, such as those mediated by parathyroid hormone-related peptide (PTHLH; MIM 168470).^36^ Normal tooth eruption proceeds through several well-defined stages including the pre-eruptive phase, involving positional adjustments of the tooth germ within the jaw; the intraosseous phase, characterised by eruption through bone; and the supra-osseous phase, wherein the tooth emerges into and adjusts within the oral cavity.^37^

## The dental follicle: a key modulator in tooth eruption

The dental follicle plays a critical role in the regulation of tooth eruption, acting as a key signalling centre that mediates bone remodelling around the developing tooth.^38^ It secretes important signalling molecules, including PTHLH, colony-stimulating factor 1 (CSF1; MIM 120420), and receptor activator of nuclear factor kappa-B ligand (RANKL; MIM 602462), all of which are essential for the recruitment and differentiation of osteoclasts.^39^ This osteoclastic activity enables the resorption of alveolar bone, clearing a path for the tooth to erupt into the oral cavity.^38^ Notably, PTHLH, produced by both the enamel organ and the dental follicle, acts through the PTH1R to mediate local bone resorption and remodelling by upregulating RANKL and downregulating osteoprotegerin (OPG; MIM 602463),^40^ and genetic variants in the *PTH1R* gene are implicated in primary failure of tooth eruption.^41^ This intricate interplay of signalling factors underscores the complex mechanisms involved in tooth eruption and highlights the essential functions of the dental follicle in this biological process.^38^

A seminal canine study strongly supports the critical role of the dental follicle in regulating tooth eruption. In an experiment where developing tooth germs were surgically removed without replacement, the dental follicles alone proceeded to erupt through the alveolar bone.^32^ In another classic study, Marks and Cahill^34^ demonstrated that when developing tooth germs were replaced with inert metal objects, these metal objects unexpectedly followed the physiological eruption pathway, provided the dental follicle remained intact. This finding underscores that the eruptive process from those stages onwards is orchestrated primarily by molecular and/or mechanical signalling from the follicular tissue rather than being actively driven by the developing tooth itself.^34^ Collectively, these observations indicate that the dental follicle functions is a critical regulatory organ, coordinating the recruitment and activity of osteoclasts and osteoblasts essential for bone remodelling and formation of the eruptive pathway.^38^ Although studies suggest that the dental epithelium may contribute to the eruptive process through signalling or structural roles,^42^^,^^43^ its presence appears to be non-essential. In other words, the dental epithelium is considered dispensable for eruption, as the fundamental signalling mechanisms mediated by the dental follicle are sufficient to initiate and direct eruption even in the absence of a tooth germ.

However, conflicting results have been reported regarding the relative importance of the dental follicle compared to the reduced enamel epithelium, based on the expression of Fam20a, a gene well known for its association with AI and failure of tooth eruption.^2^, ^3^, ^4^ The strong Fam20a expression in the reduced enamel epithelium of eruptive-phase molars, compared with its lower expression in the periodontal ligament and other areas of the dental follicle, suggests that the reduced enamel epithelium may play a more significant role than the dental follicle and periodontal ligament during this phase of eruption.^44^ However, stronger gene expression alone does not necessarily imply greater functional importance. Expression levels indicate where a gene product is actively synthesised but do not confirm its direct role without supporting functional evidence. In other words, the strong expression of Fam20a may be a ‘hint’ of the tissue’s involvement rather than a ‘proof’ of its essential function in the eruption process. It remains possible that tissues with lower Fam20a expression contribute critically through other pathways or regulatory mechanisms. Therefore, although these findings highlight the reduced enamel epithelium as a potentially important contributor to tooth eruption, further studies—such as tissue-specific functional analyses or knockout models—are needed to clarify the precise roles of Fam20a in different dental tissues.

Considering these findings, the eruption failure frequently observed in individuals with AI cannot be attributed solely to structural abnormalities of the enamel organ or defects in mineralisation. The fact that most teeth in patients with AI erupt successfully suggests that additional factors—such as intrinsic signalling deficits within individual tooth germs or disruptions in the local microenvironment—may contribute to impaired eruption. This reinforces the need to consider cellular and molecular mechanisms beyond enamel defects in the pathogenesis of eruption anomalies in AI.

It is hypothesised that pathogenic variants causing AI may disrupt amelogenesis while simultaneously impairing dental follicle signalling, thereby interfering with the eruption process. This hypothesis is biologically plausible given the shared developmental signalling pathways between the epithelium-derived ameloblasts and the mesenchyme-derived dental follicle cells. Both rely on epithelial–mesenchymal interactions mediated by signalling molecules such as BMPs, WNT, SHH and TGF-β.^38^^,^^45^^,^^46^ Disruption of these pathways in AI could therefore have pleiotropic effects—not only impairing enamel formation but also compromising the follicle’s ability to recruit osteoclasts and remodel bone. However, to date, only isolated case reports have described this overlap, and such associations may have been overlooked or underreported in clinical settings. Indeed, studies have shown that in AI patients with unerupted teeth, the dental follicle may exhibit reduced vascularity, fibrosis or poor cellular organisation, all of which suggest impaired function.^47^

A parallel can be drawn with primary failure of eruption, which highlights the critical role of PTHLH–PTH1R signalling in regulating tooth eruption, suggesting that a similar mechanism could contribute to eruption failure in some AI cases.^48^^,^^49^ Given that PTHLH is a key effector molecule in the follicle and is essential for osteoclastogenesis, genetic variants that affect its expression or signalling—even indirectly—could be sufficient to impair eruption. Importantly, a clear distinction between correlation and causation must be maintained, as some observed associations may be coincidental, multifactorial or not yet fully understood. Further research into the underlying regulatory mechanisms—including potential roles for epigenetic factors and gene–gene interactions—is essential to clarify these relationships.

In summary, the failure of tooth eruption observed in certain cases of AI may not be attributable solely to enamel structural abnormalities. Instead, it may also reflect intrinsic defects within the dental follicle, resulting from pathogenic variants in AI-associated genes that disrupt its capacity to regulate bone remodelling through molecular signalling. This hypothesis underscores the need for further investigation—particularly at the molecular and genetic levels—into the expression profiles of eruption-related genes within the dental follicles of patients with AI, as well as the development of animal models to track the early dynamics of cell signalling, differentiation and morphogenesis. A deeper understanding of these pathways could ultimately support the development of targeted therapeutic strategies for managing eruption disturbances in AI and related conditions. Moreover, elucidating the mechanisms that govern tooth eruption and movement may have translational value for improving the efficiency and predictability of orthodontic tooth movement in clinical practice.

## Conflict of interests

None declared.
